# Investigating the impact of chronic graft‑versus‑host disease on patient health‑related quality of life: perspectives from patients and carers

**DOI:** 10.1186/s12955-026-02571-3

**Published:** 2026-06-25

**Authors:** Katharina Ecsy, Luke Skinner, Martin Furze, Dawn Hart, Charlotte Sugden Heron, Amy Page, Larissa Dempsey, Adlene Adnan, Arunesh Sil, Richard D. A. Hudson, Matthew Bradberry

**Affiliations:** 1 Sanofi, Reading, UK; 2 Anthony Nolan, Royal Free Hospital, London, UK; 3 Synergy Healthcare Research, London, UK

**Keywords:** Chronic graft-versus-host disease, Quality of life, Patient-reported outcomes, Hematopoietic stem cell transplantation

## Abstract

**Background:**

Chronic graft-versus-host disease (cGvHD) is a common complication that can occur following hematopoietic stem cell transplantation. This complex, multisystemic condition often requires multiple lines of therapy to treat both the symptoms of cGvHD and the side effects associated with these treatments. Comprehensive data on the impact of cGvHD on health-related quality of life (HRQoL) of patients who have undergone multiple lines of therapy, remain limited. Greater awareness of these challenges could lead to more personalized treatment plans and inform future research.

**Methods:**

We performed a two-part study to gather qualitative and quantitative data on the experiences and HRQoL of adult patients with cGvHD who had received more than two lines of therapy. Participants took part in a virtual, in-depth interview moderated by a facilitator (qualitative interviews) and/or an online survey (quantitative survey). The qualitative interviews were conducted using a discussion guide, while the quantitative survey designed based on the responses to the qualitative interview also included the European Quality of Life 5 Dimensions 5 Level Version questionnaire.

**Results:**

The qualitative interview included 10 participants, while the quantitative survey enrolled 27. patients reported that cGvHD has a profound impact on numerous aspects of HRQoL. Treatment side effects, treatment burden, frequency of medical appointments, and the impact on daily life, finances, education, and mental health were all contributors to diminished HRQoL. Patients reported feeling unprepared for the severity and longevity of cGvHD symptoms and highlighted the need for improved communication between healthcare professionals (HCPs) treating the same patient.

**Conclusions:**

cGvHD significantly impacts the HRQoL of patients, with physical, emotional, and financial burdens. Improved education and disease awareness, personalized treatment plans, and better communication between HCPs treating the same patient are crucial to address these challenges in current care practices. Enhanced support services could alleviate the emotional and financial impact on patients, ultimately improving their overall quality of life (QoL).

**Supplementary Information:**

The online version contains supplementary material available at https://doi.org/10.1186/s12955-026-02571-3.

## Background

Chronic graft versus host disease (cGvHD) remains one of the most significant long-term complications following allogeneic hematopoietic cell transplantation (HCT), affecting approximately 30–50% of transplant survivors and can lead to long-term morbidity and mortality [1]. It is characterized by immune-mediated tissue injury (chronic inflammation and fibrosis) of multisystem organs involving the skin, oral mucosa, eyes, gastrointestinal tract, lungs, liver, and musculoskeletal system (fascia/joints) [2]. Management commonly begins with systemic corticosteroids with or without calcineurin inhibitors, but many patients become steroid refractory or steroid dependent, necessitating further systemic therapies over time. Despite multiple regulatory approvals in later lines (e.g., ruxolitinib, ibrutinib, belumosudil, axatilimab), there is neither universally accepted second-line standard nor an evidence-based consensus on a preferred second line regimen for optimal treatment strategies has not been established, and hence real-world clinical practice patterns remain variable [3, 4].

Patients who progress to two or more lines of systemic therapy represent a subgroup with disproportionate symptom burden, prolonged treatment cycling, cumulative adverse effects, substantial morbidity and high healthcare utilization, all of which can impair health related quality of life (HRQoL) [2, 3, 5]. Collectively, this literature suggests that, beyond survival, HRQoL is substantially affected by both disease manifestations and treatment toxicities. Consequently, there has been a growing shift in focus from survival alone to patient-centered/reported outcomes, particularly HRQoL. Greater awareness of such HRQoL challenges could lead to the development of more personalized treatment plans, improve communication of patients with health-care provider and inform future research efforts [6].

HRQoL is a multidimensional construct encompassing physical, psychological, emotional, and social well-being. Assessment of HRQoL in cGvHD has used a combination of generic and disease specific instruments [7]. Frequently utilized measures include the EQ-5D-5 L (generic utility) [7], short form survey (SF 36; generic profile) [8], Functional Assessment of Cancer Therapy-Bone Marrow Transplant (FACT-BMT; transplant specific) [9], and the Lee cGvHD Symptom Scale (LSS) and modified LSS (cGvHD specific) [10]. Despite the availability of various assessment instruments along with heterogenous nature of this condition, HRQoL data focused on patients with cGvHD who have progressed to ≥ 2 lines of systemic therapy remain limited. Moreover, integrating carer input as proxy perspective alongside patient reports to contextualize daily burden and care coordination challenges in routine practice. Understanding these perspectives is essential for developing holistic and patient-centered management strategies.

To address these gaps, we conducted a two-part study to gain a comprehensive overview of the experiences of patients and carers managing cGvHD after multiple treatment failures and its impact on HRQoL. Part 1 involved qualitative data collection through an in-depth, virtual interview moderated by an expert facilitator, and part 2 involved quantitative data collection through an online survey. The findings may provide valuable insights to improve supportive care and inform future clinical practice and research in cGVHD survivorship.

## Methods

United Kingdom-based adult patients and/or carers of adult patients with moderate or severe cGvHD, experiencing ongoing symptoms and having received two or more lines of therapy, were invited to participate in a virtual, in-depth interview moderated by an expert facilitator (qualitative data) and/or a 25-min online survey (quantitative data).

### Qualitative interviews

Invitations to take part in a virtual interview were disseminated by Anthony Nolan (UK registered charity and national stem cell donor register). Following screening, in-depth interviews were conducted using a discussion guide, with the patient alone (up to 60-min duration) or with the patient and their carer (up to 90-min duration). Patients and/or carers were asked the same interview questions, with carers providing responses on behalf of the person they care for. Interview questions aimed to cover lived experience of cGvHD across key domains: multi-system physical symptoms (skin, eyes, mouth, lungs, gastrointestinal/liver, genitourinary, joints/musculoskeletal, infections, fatigue/pain) and their day-to-day impact; treatment journeys (sequence of systemic and supportive therapies), side effects and experiences with extracorporeal photopheresis; healthcare burden (specialists involved, appointment frequency, travel time/costs, and unplanned care); psychological and emotional consequences; and social, financial, employment/education effects (including time off work, changes to work patterns, and interactions with welfare/benefits). Further details regarding the qualitative interview protocol and questions can be found in the Supplementary Information.

### Quantitative survey

The design of the quantitative survey was informed by responses to the qualitative interview. Invitations to take part in an online survey were publicly disseminated across the Patients and Families Forum and Facebook page of Anthony Nolan, a UK registered charity and stem cell donor registry. The survey questions comprised structured modules on demographics/clinical background and awareness of cGvHD before/at diagnosis; a comprehensive multi-organ symptom checklist with frequency and impact-on-daily-life ratings; treatment exposure and treatment-related side effects (with impact ratings); detailed ECP experience (uptake, cadence, duration, reasons for discontinuation, practical/financial implications); healthcare utilisation (specialists seen, appointment cadence, unplanned/emergency visits) and perceptions of care coordination; and broad daily-life/HRQoL impacts (mobility, independence, self-care, sleep, relationships, appearance, hobbies, aspirations/planning, ability to work/study, finances, and mental health). Generic HRQoL was captured using the European Quality of Life 5 Dimensions 5 Level Version (EQ-5D-5 L) questionnaire, which is a validated and internationally recognized psychometric tool to measure HRQoL across multiple disease states [11]. Further details regarding the quantitative survey protocol and questions can be found in the Supplementary Information.

### Statistical analysis

Qualitative interview data were transcribed and summarized using reflexive thematic analysis following Braun and Clarke’s framework (familiarization; inductive coding; theme generation; review; definition; reporting). Quantitative survey data were analyzed descriptively, reporting counts and percentages for categorical variables in line with recommended reporting practices for observational studies (STROBE). Distributions of continuous variables were examined using histograms/Q-Q plots; when formal assessment of normality was informative for summary choice or any exploratory comparisons, we applied the Shapiro-Wilk test, given its favorable performance in small-to-moderate samples. HRQoL was summarized using the EQ-5D-5 L: we report domain-level profiles and utility indices. Because the cohort is UK-based, primary utility estimates were derived using the cross-walk mapping of EQ-5D-5 L value set in accordance with the National Institute for Health and Care Excellence (NICE) for England. Missing data were handled by available-case analysis with the denominator explicitly reported for each item; no imputation was performed given the exploratory descriptive objective of this study that is PRO research. SPSS Statistics v29 (IBM) was used for data management and analysis. Figures were created in R (version 4.x) and RStudio.

## Results

### Participant characteristics

Ten participants (eight patients and two carers) were enrolled in the virtual qualitative interview. Twenty-seven participants (seventeen patients; eight current carers; two previous carers) were enrolled in the online quantitative survey. Participant characteristics and demographics are summarized in Table 1.

**Table 1:** Participant characteristics and demographics

**Respondent type**,* n* (%)				
		**Respondents*** N* = 10		
**a. Qualitative interviews**				
Patient		8 (80)		
Carer		2 (20)		
**Sex**, ***n***				
Female		6 (60)		
Male		4 (40)		
		**Patients* n*** **= 8**		
**Age**, ***n (%)***				
< 50 years		4 (50)		
≥ 50 years		4 (50)		
**Location**, ***n (%)***				
England (any region)		7 (87.5)		
Devolved nations (Wales/Scotland)		1 (12.5)		
**Previous conditions**, ***n (%)***				
Acute myelogenous leukemia		3 (37.5)		
Myelodysplastic syndromes		3 (37.5)		
Myelofibrosis		1 (12.5)		
Acute lymphoblastic leukemia		1 (12.5)		
**b. Quantitative survey**				
	**Total patients** ^**a**^ *N* = 27		**Patients** **(*n*** **= 17)**	**Carers** ^**b**^ ***n*** **= 10**
**Sex**, ***n*** **(%)**				
Male	21 (78)		12 (71)	3 (30)
Female	6 (22)		5 (29)	7 (70)
**Ethnicity**, ***n*** **(%)**				
White	26 (96)		16 (94)	10 (100)
Asian/Asian British	1 (4)		1 (6)	0 (0)
**Age in years**, ***n*** **(%)**				
18–44	11 (41)		6 (35)	5 (50)
45–60	9 (33)		7 (41)	2 (20)
≥ 61	7 (20)		4 (24)	3 (30)
**Location**, ***n*** **(%)**				
England (any region)	21 (78)		13 (76)	8 (80)
Devolved nations (Scotland/Wales)	6 (22)		4 (24)	2 (20)^c^
**Employment status**, ***n*** **(%)**				
Working (full-/part-time)	11 (41)		5 (29)	6 (60)
Not working^‡^	16 (59)		12 (71)	4 (40)
**Household income**, ***n*** **(%)**				
≤ £20,000	6 (22)		3 (18)	3 (30)
£20,001 - £40,000	8 (30)		4 (24)	4 (40)
> £40,000	10 (37)		8 (47)	2 (20)
Not reported^§^	3 (11)		2 (12)	1 (10)

^a^Calculation based on responses from 17 patients and 10 carers who were asked about the patients they cared for

^b^Carer column based on responses when carers were asked about themselves. ^c^Only one carer, who did not live in the same household as the person they cared for, was asked about their location

‡ Not working includes retired, unable to work due to illness, student, unemployed, and stay-at-home

§ “Not reported” includes “not sure” and “prefer not to say”

### Awareness of cGvHD pre-diagnosis

In the qualitative interviews, respondents reported that patients had little awareness of cGvHD prior to their stem cell transplant, as their primary focus was finding a suitable donor match. Most patients assumed that if they developed GvHD, it would likely be acute, temporary, and easily treatable. One patient stated “I wasn’t prepared for the severity and impact of chronic… I was told you could treat it with cream, nip it in the bud.” Post-transplant, patients felt there was a lack of clarity around when to start worrying about symptoms, leaving them feeling unprepared for the debilitating impact of cGvHD. The quantitative survey uncovered that upon receiving a cGvHD diagnosis, 92% of patients did not appreciate how long they could experience cGvHD symptoms for, and only 22% were fully informed about all treatment options available. Despite these challenges, 63% of patients still expected treatments to be effective in managing their condition.

### Physical impact of cGvHD and treatment on HRQoL

#### Symptoms and treatments

**Fig. 1:**
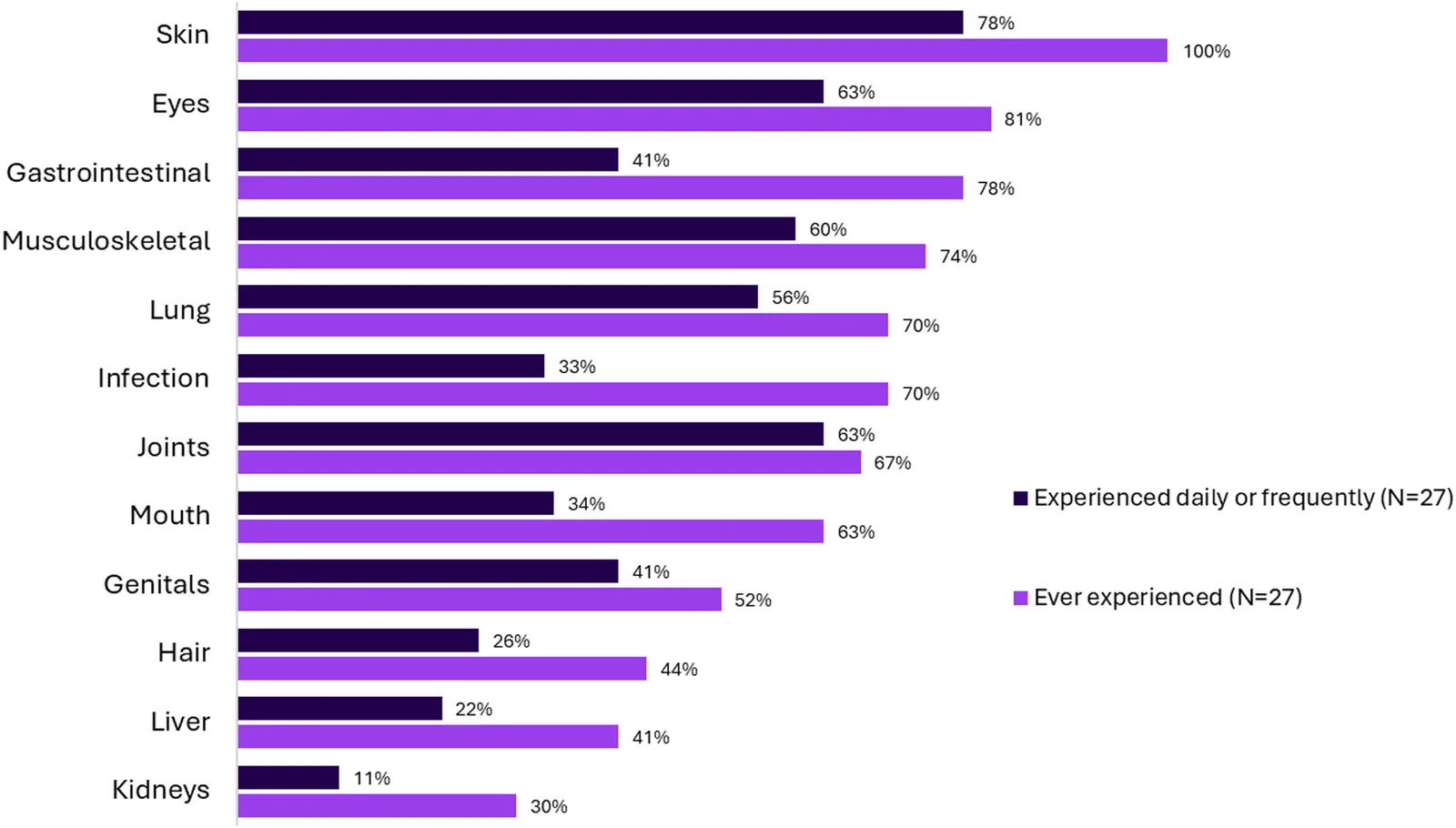
Symptoms of cGvHD experienced by patients (quantitative survey; *N* = 27 [patients, *n* = 17; carers, *n* = 10]). Q9: Which of the following physical symptoms have you ever experienced as a result of cGvHD? [Patient]. Which of the following physical symptoms has (did) the person you care(d) for ever experience(d) as a result of cGvHD? [Carer]. Q10-Q10i: How often is (was) the symptom experienced? [Patients] How often is (was) the symptom experienced? [Carers]. cGvHD, chronic graft-versus-host disease

**Fig. 2:**
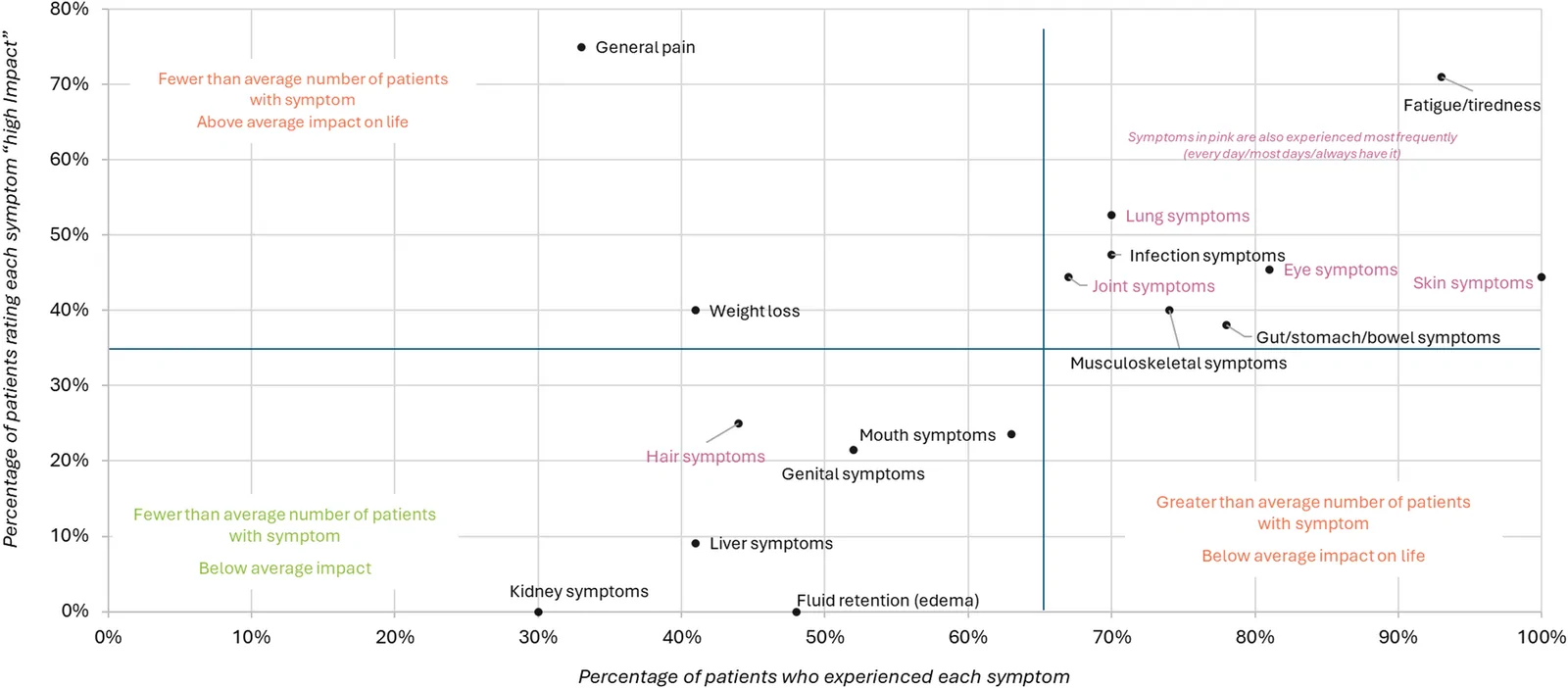
Symptoms of cGvHD ever experienced by patients and impact on daily life (quantitative survey; *N* = 27 [patients, *n* = 17; carers, *n* = 10]). Graph split into four quadrants to visualize percentage of patients experiencing each symptom and whether that symptom has a high impact on their day-to-day life. Q16: For each of the side effects you experienced as a result of treatment, how did this impact on your day-to-day life? [Patients]. For each of the side effects they experienced as a result of treatment, how has (did) this impact(ed) their day-to-day life? [Carers]. cGvHD, chronic graft-versus-host disease

In the qualitative interviews, cGvHD was reported to restrict the daily lives of patients and carers, affecting mobility, independence, physical appearance, relationships, hobbies, exercise, diet, and sleep. Patients described the impact of cGvHD symptoms on their quality of life (QoL), with one patient stating “It’s tough, there’s no end in sight. It’s just relentless…I have to keep going for the kids, but sometimes you feel like you are going mad.” A wide range of physical symptoms that affect numerous organs and vary in severity were reported by patients and their carers across both studies. The most commonly reported symptoms experienced among patients in the quantitative survey involved the skin (100%), eyes (81%), and gastrointestinal system (78%) (Fig. 1), with most reporting a daily or frequent occurrence. Symptoms with higher prevalence generally clustered in upper right quadrant, indicating both frequent occurrence and greater impact on daily life. Skin, eye, lung and joint symptoms were associated with particularly high daily burden, whereas less frequently reported symptoms such as kidney and liver involvement showed comparatively lower overall impact (see Fig. 2).

#### Treatment related side effects and treatment burden

**Fig. 3:**
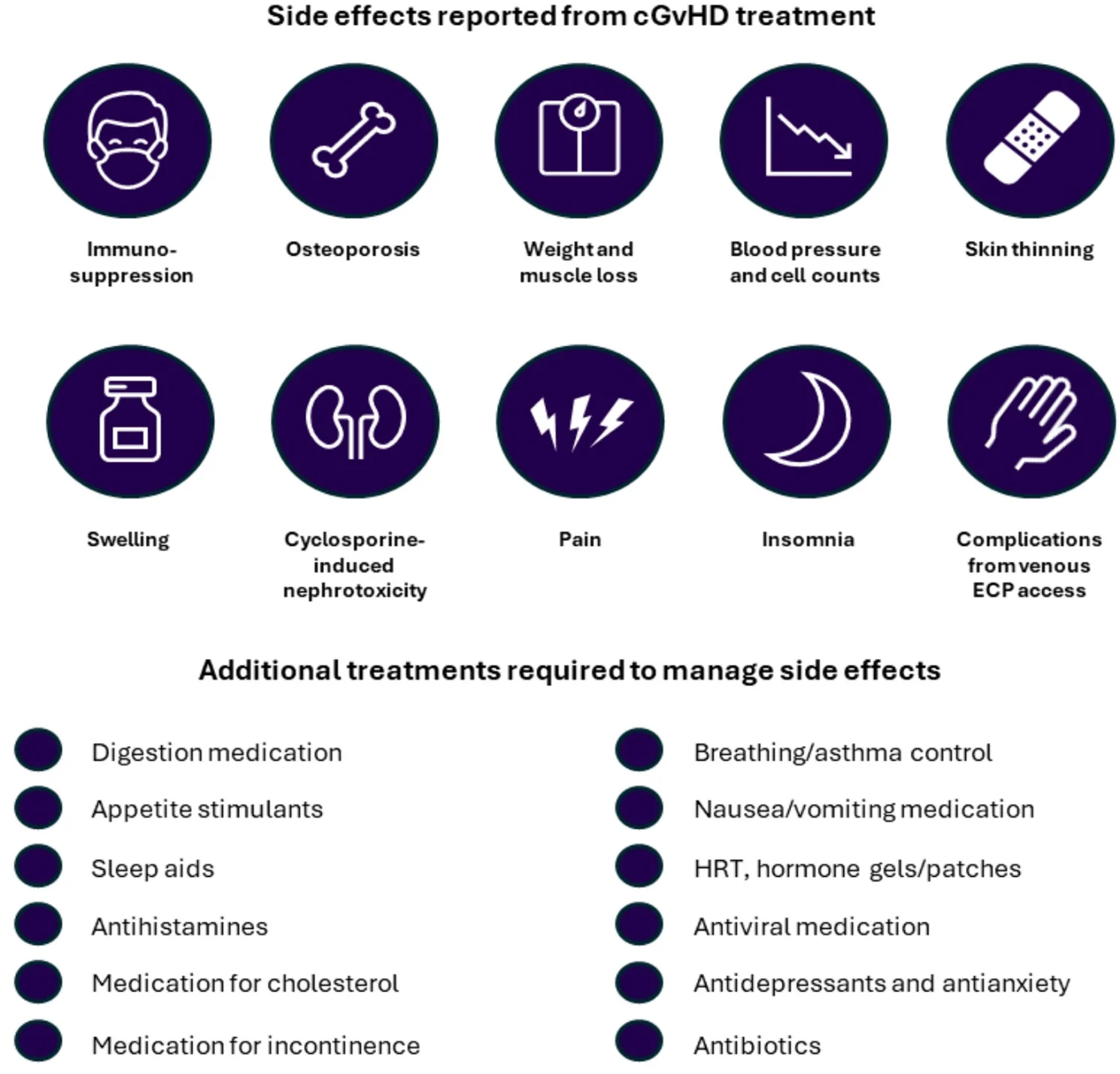
Side effects and complications of cGvHD treatments (qualitative interviews, *N* = 10 [patients, *n* = 8; carers, *n* = 2]). cGvHD, chronic graft-versus-host disease; ECP, extracorporeal photopheresis; HRT, hormone replacement therapy

Treatments for cGvHD often lead to side effects and complications, necessitating further treatments as reported by patients and carers in both studies, with one patient expressing that “You take one for this, one for that, this one has a side effect, so you take that.” A summary of the side effects and complications highlighted by participants in the qualitative interviews is presented in Fig. 3. Patients in the qualitative interviews reported feeling regularly overwhelmed by side effects from multiple medications. Additionally, many respondents had to organize their lives around medications, significantly limiting travel and social life. One patient shared their experience of setting alarms for every 2 h to remind them to take their medications, and another reported taking 12 different medications a day.

In quantitative survey, treatment related side effects were reported by 92% of surveyed patients, most commonly general (78%) and gastrointestinal (78%) effects, such as fatigue (67%), moon face (56%), edema (44%), weight gain (41%), constipation (37%), and diarrhea (33%). Side effects affecting the skin, eyes, lungs, joints/bone, infections, and mouth were associated with greater daily life impact. In this survey, 61% had received ≥ 3 systemic therapies, 78% had used steroids, 74% immunosuppressants, and 40% were taking ≥ 6 concurrent treatments. Use of non-systemic therapies (e.g., painkillers and topical agents, each 70%) and additional supportive medicines were widespread (89%), particularly antibiotics (74%), antivirals (67%), and symptom management agents. A summary of these results is provided in Table 2.

**Table 2:** Summary of treatment-related side effects and treatment burden among patients in the quantitative Survey (*N* = 27)

Domain	Findings	% of patients
Any treatment-related side effects	Reported experiencing ≥ 1 side effect	**92%**
General side effects	Fatigue; moon-face; edema	78% (Fatigue 67%; Moon-face 56%; Edema 44%)
Gastrointestinal side effects	Weight gain; constipation; diarrhoea	78% (Weight gain 41%; Constipation 37%; Diarrhoea 33%)
High-impact side-effect domains	Skin, eyes, lungs, joints/bone, infections, mouth	High impact reported; percentages shown in Fig. 2
Sensory system side effects	Less common but high daily-life impact	< 50%
Systemic treatment burden	≥ 3 systemic therapies received	**61%**
	Ever used steroids	**78%**
	Ever used immunosuppressants	**74%**
	Taking ≥ 6 treatments concurrently	**40%**
	Ever used other systemic treatments (e.g., IV/oral meds)	**81%**
Non-systemic treatments	Painkillers; creams/ointments	**70%** each
	Eye drops	**59%**
	Inhalers	**44%**
Additional supportive medicines	Required ≥ 1 supportive medicine	**89%**
	Antibiotics	**74%**
	Antivirals	**67%**
	Medicines for digestion	**44%**
	Medicines for breathing	**41%**
	Nausea medicines	**37%**
	Osteoporosis medicines	**33%**
	Depression/anxiety medicines	**30%**

### Quality of care impact of cGvHD on HRQoL

Findings from this study indicate that attending multiple, regular medical appointments adds an additional burden for patients and their carers. One patient who regularly visited hospital to receive treatment with extracorporeal photopheresis stated “…I was so anxious every time I went to the hospital. Getting there, getting it done. It was stressful.”

According to the quantitative survey, one third of patients attended an appointment for cGvHD several times a month, with 37% experiencing unplanned hospital visits monthly or a few times a year. Patients interacted with an average of five different healthcare professionals (HCPs) in relation to their cGvHD, including hematologists (89%), dermatologists (70%), and ophthalmologists (59%).

Patients in the qualitative interviews found it frustrating to visit numerous specialists and repeat their medical history each time, noting a lack of communication between different departments. For example, patients reported that they were prescribed contraindicated drugs, resulting in patients feeling obligated to become “experts” themselves.

Some patients in the qualitative interviews also felt that their previous medical condition was given insufficient consideration when they developed cGvHD. Most patients in the quantitative survey (66%) expressed the need for improved communication between HCPs involved in managing their cGvHD, with 48% preferring to have one doctor overseeing their cGvHD management.

### Emotional impact of cGvHD and treatment on HRQoL

**Fig. 4:**
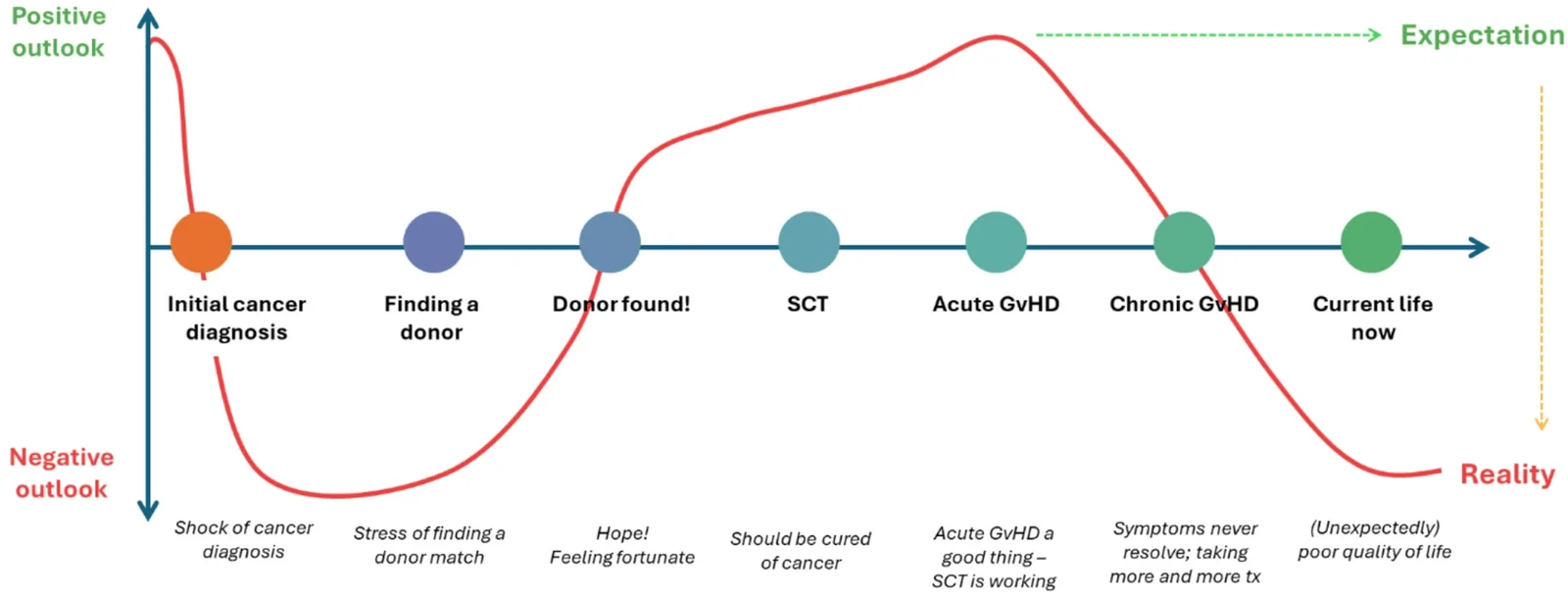
The emotional journey experienced by patients from cancer diagnosis to living with cGvHD (qualitative interviews, *N* = 10 [patients, *n* = 8; carers, *n* = 2]). cGvHD, chronic graft-versus-host disease; GvHD, graft-versus-host disease; SCT, stem cell transplant; tx, treatment

Patients in the qualitative interviews reported experiencing a variety of emotions from the moment of their initial cancer diagnosis to their current life. Patients felt negative following the shock of their cancer diagnosis and the stress associated with finding a suitable stem cell donor. Soon after, patients’ outlook improved when a stem cell donor was found, with this sense of hopefulness continuing following transplantation. Unfortunately, a cGvHD diagnosis, along with worsening symptoms and the high treatment burden, contributed to a resurgence of negative emotions (see Fig. 4). Some patients ultimately felt apathetic toward tracking their disease management.

**Fig. 5:**
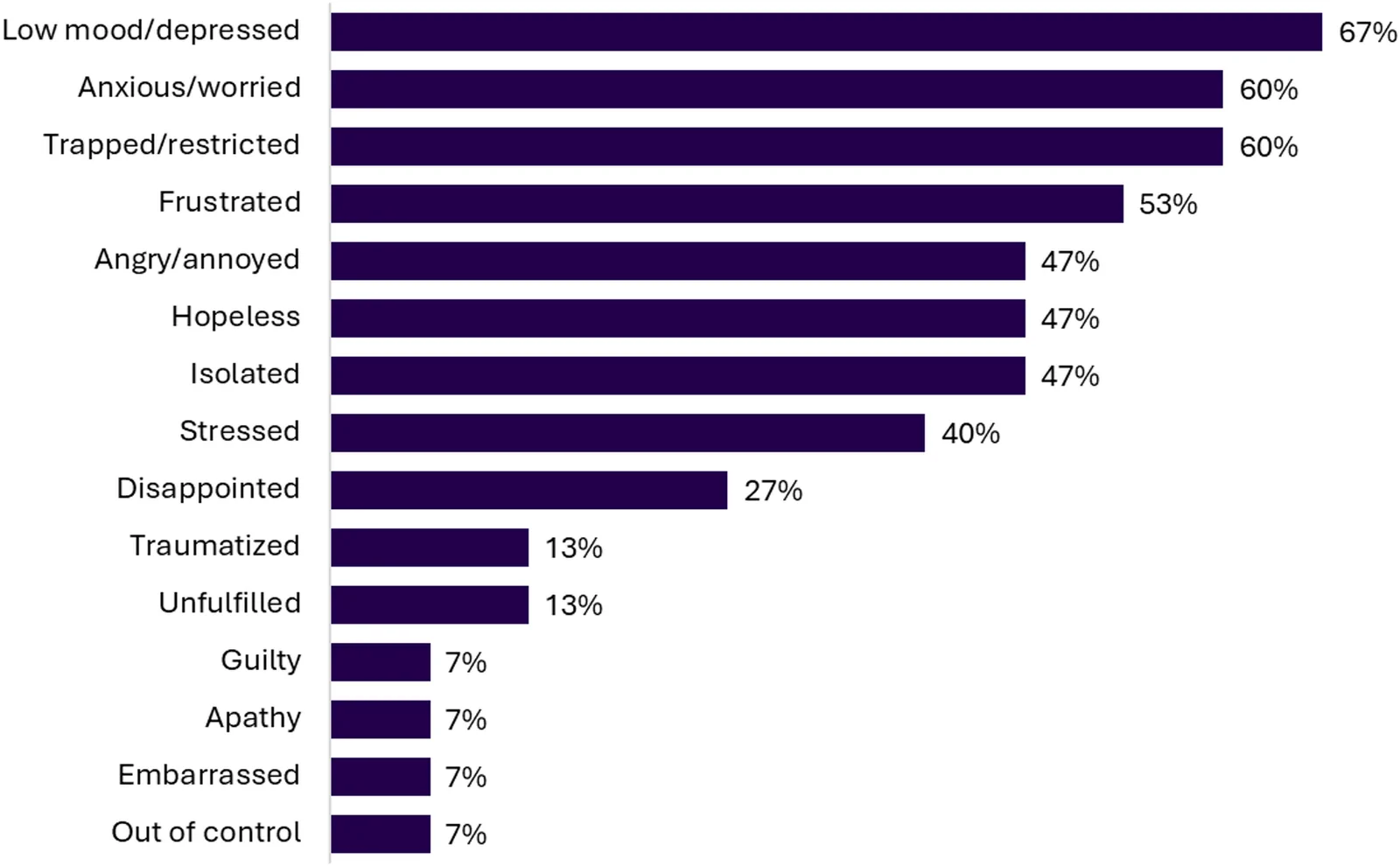
Emotions experienced in connection with cGvHD (quantitative survey; *N* = 27 [patients, *n* = 17; carers, *n* = 10]). Q32: Which of the following emotions, if any, have you experienced in connection with your cGvHD? [Patient]. cGvHD, chronic graft-versus-host disease

During the qualitative interviews, patients and carers highlighted the numerous psychological consequences of cGvHD on their lives, such as heightened anxiety, diminished enjoyment of hobbies or interests, decreased confidence, and strained relationships. The results of the quantitative survey showed that 69% of patients found that cGvHD impacted their mental health, and 78% felt they were a burden to others due to their condition. The wide range of emotional experiences reported in relation to cGvHD are summarized in Fig. 5, the most common of which was low mood/depression, reported by 67% of patients.

The results of the qualitative interviews revealed that managing the symptoms of cGvHD and dealing with the side effects of treatments elevated health anxiety among both patients and their carers. Patients reported that consulting a range of specialists and attending multiple, regular appointments took an emotional toll and caused them to feel like a burden when relying on family for transportation.

Increased infection risk due to immunosuppressive therapy led patients and carers in the qualitative interviews to experience isolation and heightened health anxiety. Some patients became housebound as a precaution due to fear of infection when venturing outside. This anxiety extended to friends and family who shared concerns about potentially transmitting infections to the patient. One patient stated “It has had a huge impact on our relationships. My daughter and sister, they get really anxious about visiting us in case we catch something from them. They would feel responsible.”

Mental health support was offered to only 52% of patients participating in the quantitative survey, while 35% were not offered any mental health support. Many patients and carers (74%) felt that people with cGvHD require more emotional support than currently available. All carers reported feeling anxious or worried when caring for someone with cGvHD and expressed the need for additional emotional support.

### Financial/educational impact of cGvHD on HRQoL

Patients and carers reported experiencing a financial impact associated with cGvHD, including reduced income and increased financial pressure due to leaving their jobs. The quantitative survey showed that 41% of patients have taken time off work due to cGvHD, while 37% stopped work completely. One patient in the qualitative interviews stated “I was a teacher, but I just had to give up due to the infection risk and I was too breathless. Any infection could take three, four weeks to recover from. So, I had to stop.”

Both studies found that cGvHD prevented patients and carers from pursuing their goals. Only a few patients in the qualitative interviews were still hopeful for a return to work or education in the future. One patient reported “It stopped all my career plans” and “I planned a gap year after the transplant, then I was going to do teacher training. Of course, I couldn’t do that.”

All carers in the quantitative survey reported that their aspirations for the future were impacted by caring for someone with cGvHD. The majority (90%) felt that caring for someone with cGvHD had a moderate to high impact on their ability to plan for the future or work/study and 60% said their finances were affected.

Hidden costs associated with cGvHD, such as travel expenses, catering to new dietary needs, and non-prescription medications were also reported in the qualitative interviews.

### Overall daily impact of cGvHD on HRQoL

**Fig. 6:**
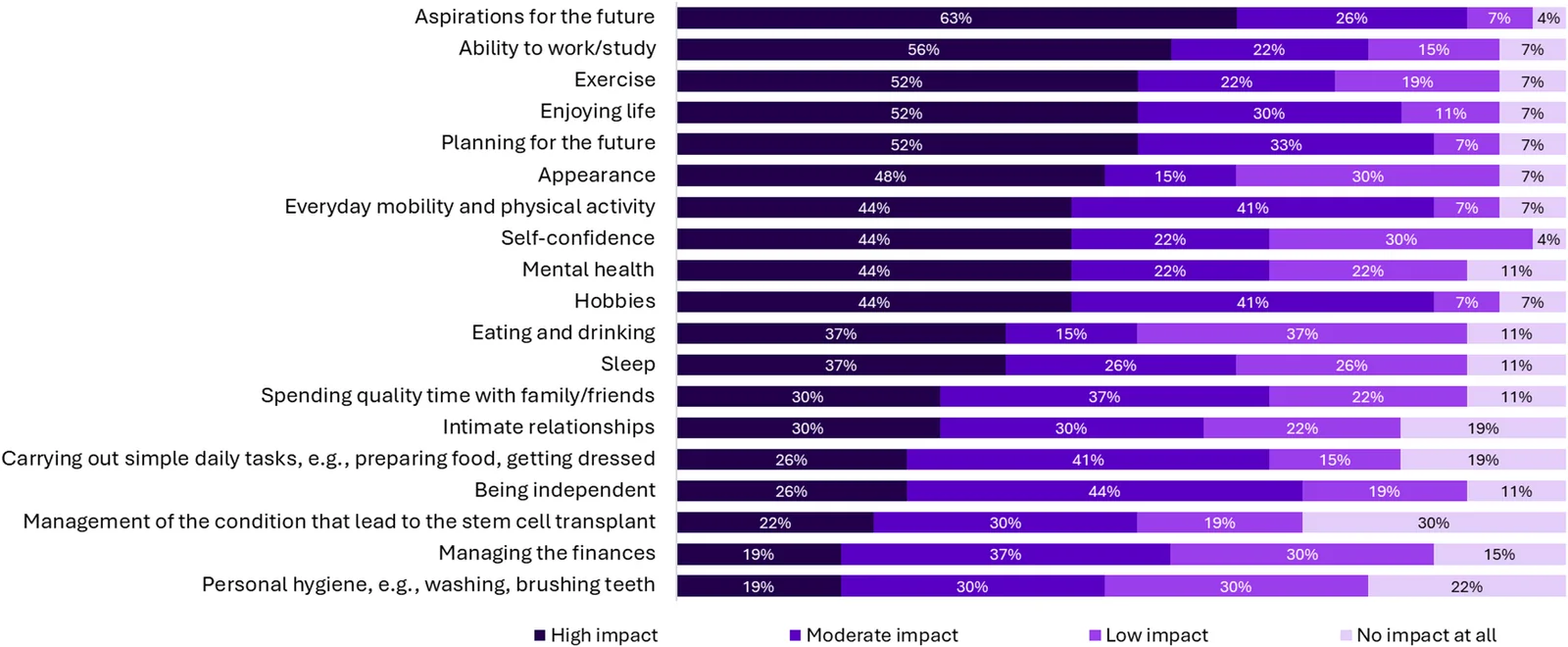
The daily impact of cGvHD on patient HRQoL (quantitative survey; *N* = 27 [patients, *n* = 17; carers, *n* = 10]). Due to rounding of percentages, the bars may not all add up to 100. Q28: Please tell us how much impact cGvHD has on each of these aspects of your daily life [Patient]. Please tell us how much impact cGvHD has on each of these aspects of the daily life of the person you care(d) for [Carer]. cGvHD, chronic graft-versus-host disease; HRQoL, health-related quality of life

**Fig. 7:**
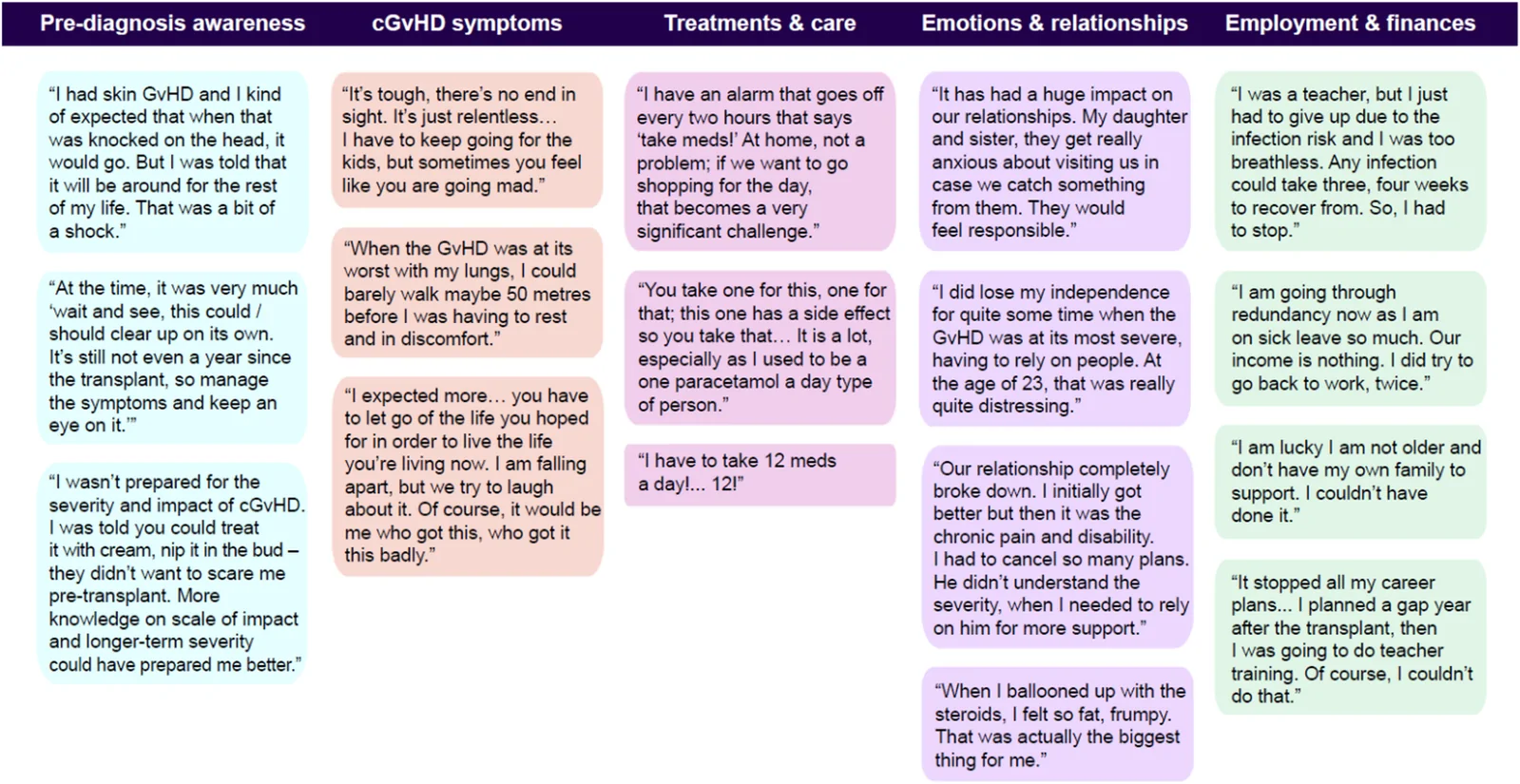
The impact of cGvHD on patient HRQoL: Key patient insights (qualitative interviews, *N* = 10 [patients, *n* = 8; carers, *n* = 2]). cGvHD, chronic graft-versus-host disease; GvHD, graft-versus-host disease; HRQoL, health-related quality of life

According to the quantitative survey respondents, the greatest impact of cGvHD on patient HRQoL was on aspirations for the future (89% reported a moderate to high impact), followed by planning for the future (85%), mobility and physical activity (85%), and hobbies (85%) (see Fig. 6). Furthermore, EQ-5D-5 L questionnaires found that 84% of patients experienced problems with pain or discomfort, and more than two-thirds experienced issues with mobility, usual activities, and anxiety or depression. For qualitative interview respondents, a summary of key insights regarding the impact of cGvHD on HRQoL is presented in Fig. 7.

## Discussion

cGvHD remains one of the most challenging late complications following allogeneic HCT, with reported incidence ranging from 30% to 70% depending on donor source, conditioning intensity, and prophylaxis strategy. This multisystem disorder continues to impose extensive morbidity, prolonged healthcare utilization, and high treatment burden, making it a leading contributor to impair HRQoL among transplant survivors [12, 13]. In this context, the present two-part study sought to address persistent gaps in real-world understanding by examining the multifaceted impact of cGvHD on the HRQoL of patients, combining both qualitative insights with targeted quantitative assessment.

This study highlighted a pressing concern: there is limited awareness and misconceptions about cGvHD both prior to and at the time of diagnosis. As such, there is a significant need for improved approaches to educating patients and carers prior to and throughout their transplant journey. This may support timely diagnosis of cGvHD [1], while also ensuring patients feel more prepared and able to come to terms with a diagnosis of cGvHD. Strengthening pre- and post-transplant communication may therefore represent an important avenue to reduce uncertainty, improve patient readiness, and facilitate earlier diagnosis [12].

The study also revealed the extensive symptom burden experienced by patients with cGvHD, with a range of organ-specific manifestations reported at varying severity levels. Consistent with the heterogeneity described in recent literature [2, 3, 5], the study also revealed the extensive symptom burden experienced by patients with cGvHD across numerous organ systems, including dermatologic, ocular, gastrointestinal, pulmonary, and musculoskeletal manifestations. Fatigue, reported by 93% of patients, emerged as one of the most prevalent and burdensome symptoms. This aligns with evidence showing that fatigue, pain, and musculoskeletal limitations are among the strongest determinants of reduced physical functioning and HRQoL in cGvHD [14, 15]. Symptoms can impair mobility, increase infection risk, and generally impact day-to-day activities of patients, further depleting HRQoL. Indeed, the fact that most patients were unprepared for the longevity of cGvHD had the potential to exacerbate the distress experienced even further. The findings of present study align with previous reports demonstrating substantial long term symptom burden and functional impairment in individuals living with cGvHD [16, 17].

The requirement for multiple treatments with side effects, along with frequent medical appointments can be overwhelming for both patients and carers, contributing to emotional distress and reduced QoL. Patients in this study frequently reported exposure to multiple systemic therapies, with 61% having received ≥ 3 treatment lines and many taking ≥ 6 medications concurrently. The cumulative burden of polypharmacy, including corticosteroids, immunosuppressants, and repeated systemic and topical agents, placed notable practical strain on patients, exacerbated by treatment-related side effects and the need for rigid medication schedules. The burden of symptoms and treatment side effects reported in this study suggest the importance of multidisciplinary teams in cGvHD management [18, 19].

Beyond physical symptoms and treatment complexity, our findings emphasized the challenges in care coordination. Patients noted a perceived lack of communication between departments managing their care, which increases patient burden and indicates a need for improved collaboration. It is also important that HCPs and specialists outside of transplant and late effects teams have adequate knowledge of cGvHD to ensure accurate and timely referrals, which may help to improve patient outcomes. To be effective, management of cGvHD symptoms must take a personalized approach that considers the varying symptoms among patients. Indeed, the application of personalized treatment plans has been highlighted as a priority within the cGvHD field [20]. Improving education of multidisciplinary teams and patients around risk factors and symptoms, including awareness and identification of cGvHD early in the disease course, could be transformative [13]. Our results build on previous work by emphasizing the essential role of coordinated, multidisciplinary teams in managing cGvHD, Specifically, the patient-reported burden due to a lack of communication between departments corroborates recent findings that call for enhanced collaborative practices [21], while the call for tailored symptom management supports shift toward personalized, biomarker-guided care [22].

The emotional and psychological impact of cGvHD was prominent throughout both qualitative and quantitative findings of this study. The present study reported that over two-thirds of patients experienced mental health challenges due to cGvHD. However, in previous study, one-third of patients with cGvHD reported clinically significant symptoms of depression or anxiety [23], which is lower than the proportion observed in our cohort. The higher prevalence identified in our study may reflect the greater disease severity and the predominance of patients receiving multiple lines of treatment. Moreover, these findings are unsurprising considering how physical symptoms of cGvHD and health anxiety can lead to feelings of hopelessness and isolation. The unpredictable course of cGvHD, compounded by chronic pain, treatment toxicity, and social withdrawal due to infection precautions, created significant psychological distress. These results are aligned with systemic review and metanalysis showing that psychiatric symptoms, particularly anxiety and depression, are well-established in cGvHD following HCT [24]. Better incorporation of support services into treatment plans may help to alleviate some of the emotional impact on HRQoL experienced by patients, as well as their carers who are often overlooked. As emphasized by El-Jawahri [25], supportive care should include monitoring HRQoL and symptom burden, routine screening for psychological manifestations, implementation of therapeutic approaches to treat mental health conditions, involvement of a multidisciplinary team for cGvHD symptom management, and prevention and management of infection complications. Further research into cGvHD and how it can impact mental health is critical to improve understanding and better inform approaches to supporting patients and carers. Given the negative impact that psychological distress can have on key clinical outcomes in patients with cGvHD [26], it is crucial to address the psychological needs of patients as a priority, and thus, potentially minimize the effects of cGvHD on overall well-being and QoL.

cGvHD impacts other areas of the lives of patients and carers, such as work, family, finances, and education. Similar to the findings of Yu et al. [27], this study found that cGvHD significantly impacted patients’ ability to work, with many having to leave their jobs. Given that the stress of experiencing financial challenges may further increase the burden on mental health [28], it is crucial that these financial pressures on patients and their carers are somehow addressed during treatment. According to US cohorts, only 27.7% of individuals with chronic cGvHD left a job, and financial hardship is closely associated with declines in HRQoL and psychosocial functioning [29]. Further, consistent with prior Yu et al. [27] who reported that 33.8% of patients had left their job due to cGvHD, more than one-third of patients in our study reported having stopped work entirely, with many citing health deterioration or infection risk as key factors. More awareness should be drawn to “hidden costs” such as travel expenses, catering to new dietary needs (dietary modifications), non-prescription medications, and additional care expenses, which further contributed to financial strain for both patients and carers [28, 30].

Finally, this study has several strengths and limitations that should be considered when interpreting the findings. Methodologically, this study employed a mixed-methods design in which qualitative findings shaped the structure and content of the subsequent quantitative survey, and both patients and carers (as proxies) were included to capture complementary perspectives while living with cGvHD subject. The structured symptom and treatment modules, alongside the validated generic HRQoL tool (EQ-5D-5 L), provided a broad view of disease burden, treatment exposure/side effects, care coordination, and daily life impacts. At the same time, several limitations warrant consideration. First, the sample sizes for both the interview and survey components were relatively small, which limits the generalizability of findings and reduces the precision of quantitative estimates, particularly for EQ-5D-5 L utilities, that generally benefit from larger datasets. Second, the cross-sectional, self-reported nature of data collection introduces potential reporting biases thereby precludes causal inference and sometimes proxy responses from carers, although informative for real-world burden, may not always align with patient self-perception. Third, the UK-based convenience sample may not fully represent the diversity of individuals living with cGvHD. Hence, future research should be needed to validate these findings in broader and more diverse cohorts, incorporate disease specific instruments, and prospectively link the patient and carer reported outcomes which could be addressed in larger, multi-center studies.

## Conclusion

This study demonstrates that cGvHD notably affects the HRQoL of patients who have experienced multiple treatment failures. The treatment burden, side effects, medical appointments, impact on daily life, and mental health challenges associated with cGvHD can all contribute to a diminished HRQoL for patients. The financial burden and inability to pursue work or education was also reported as a challenge associated with caring for someone with cGvHD. Addressing these factors and providing support for individuals according to their needs is crucial to improve HRQoL and alleviate the multifaceted burden of cGvHD.

## Supplementary Information

Below is the link to the electronic supplementary material.

Supplementary Material 1

## Data Availability

The data that support the findings of this study are held by Synergy Healthcare Research, but restrictions apply to the availability of these data. The dataset contains Special Category Personal Data, as categorized under data protection law, and is health data from a small patient population, and therefore, individual participants may be identifiable from the raw data. The datasets generated during the current study are, therefore, not publicly available; however, summarized, aggregated data may be available from the authors upon reasonable request and with permission of Sanofi.
