# Supplementary material for: Investigating the impact of chronic graft‑versus‑host disease on patient health‑related quality of life: perspectives from patients and carers
Source: Health Qual Life Outcomes. 2026 Jun 25;24:117. doi: 10.1186/s12955-026-02571-3 (PMC13560328; doi:10.1186/s12955-026-02571-3)
Supplement: Supplementary file 1 [file 12955_2026_2571_MOESM1_ESM.pdf]

Supplementary Information

This appendix has been provided by the authors to provide additional information relating to the methodology of their study.

Ecsy K, et al. Investigating the impact of chronic graft-versus-host disease on patient and carer health-related quality of life.

Contents

Qualitative interview questions

…… 2

Quantitative survey questions

……7

Qualitative interview questions

Background

- First of all, please could you tell me a little about yourself such as your age, your family, what you do or did for a living, and what you do in your spare time?
- Carers: And please could you tell me about the person who had the stem cell transplant that you've been looking after? Check relationship for subsequent reference
- Please can you tell me how long ago you (carers: the person you care for) had your donor stem cell transplant? If more than one stem cell transplant, confirm timescales for each; ensure capture what condition led to having a stem cell transplant

Experience of living with cGvHD

- Please could you describe in your own words what 'chronic graft-versus-host disease' (cGvHD) is? Explore respondent terminology to inform design of subsequent online survey wording
- Please can you tell me about any physical symptoms you experience due to cGvHD?
    - Which symptoms or effects have you experienced due any treatments given for cGvHD? [Interview to distinguish clearly where possible where symptoms or effects experienced are as a result of cGvHD or treatment for cGvHD]
- Interviewer to allow for spontaneous discussion of symptoms, then use below list to prompt experience of symptoms in any areas not already discussed:

Skin (e.g. rash, raised or discoloured areas, skin thickening or tightening)
Gut/stomach/bowel or (e.g. difficulty swallowing, nausea, vomiting, diarrhoea, weight loss)
Liver (e.g. abdominal swelling, yellowing skin or eyes)
Lungs (e.g. shortness of breath, changes on chest X-ray)
Mouth (e.g. dry mouth, white patches, taste changes)
Eyes (e.g. dry eyes, vision changes)

Genitals (women: e.g. vaginal dryness or painful intercourse; men: narrowing and/or scarring of the urethra, itching or scarring on the penis and scrotum, irritation of the penis)
Joints (e.g. decreased range of motion in joints or tightness in joints)
General fatigue, muscle weakness, or pain
Hair (body/scalp e.g. hair loss or changes in distribution, consistency, and colour)
Repeated infections

- Which other physical symptoms or effects of cGvHD, if any, have you previously experienced?
- Which other physical symptoms or effects have you previously experienced due to any treatments given for cGvHD?
- How do the physical symptoms and other effects of cGvHD or its treatment currently impact your day-to-day life? How, if at all, does this currently limit what you do?
- What treatments (if any) are you currently being prescribed for this condition? Please can you talk me through the sequence of any previous treatments you have previously been prescribed for this condition?
    - Ask if not mentioned: Have you received extracorporeal photopheresis (ECP) to treat cGvHD (a procedure where blood is taken and white blood cells are separated, combined with a drug, and exposed to ultraviolet light before being reinfused)? If ECP received, ask when this was initiated relative to other treatments and the number of procedures carried out
- If not fully captured: How does the treatment you are currently receiving for cGvHD currently impact your day-to-day life? Your overall quality of life? How, if at all, does it limit what you do?
- In what other ways has this condition, or its treatment, previously impacted on your day-to-day life, your overall quality of life, or limited what you've been able to do? Inquire for effects caused by disease versus caused by treatments
- How, if at all, has cGvHD or any treatments for it affected:
    - Your appearance

- Your sleep
- Your diet (as a result of any effects of cGvHD on eating/retaining food)

- How, if at all, has cGvHD and any treatment for it affected the treatment of the condition which required you to have the SCT or any other health conditions? Explore in more detail any consequences of not being able to take other treatments/impact of worsening other health conditions
- What side effects have you experienced with each of the treatments you've been prescribed for cGvHD? If time permits, for each treatment: how have the side effects of this treatment impacted on your life?
- How, if at all, has cGvHD, or its treatment, affected how frequently you need to see a healthcare professional? What impact has this had on your life, day-to-day and overall?
    - What amount of travel time and cost is involved in your care? What impact, if any, does this have on your life?
    - For patients who have received ECP, ask: what impact, if any, has ECP treatment had on your life – e.g. travel time, cost, and any impact on work or family?
- Please can you tell me about the psychological or emotional impact of living with cGvHD? Understand fully:
    - Impact on the individual's mental health (e.g. anxiety and depression)
    - Impact of the onset of GvHD after the recovery from stem cell transplant
    - Impact of need to travel for treatment
- Please can you tell me about the impact of living with cGvHD on:
    - Social activities?
    - Family life?
    - Education/career, and any impact on wellbeing of not partaking in this?
    - Sexual health?
- Please can you tell me about the financial impact of living with cGvHD?
    - Impact on the individual's employment/loss of income
    - Impact on educational or other opportunities
    - Experience of the welfare and benefits system (e.g. claims for Personal Independence Payment, Employment and Support Allowance, Carer's Allowance)

    - The cost of travel for appointments
    - Impact on life insurance/costs
    - Discrimination from work due to taking time off for treatment/appointments
- If not already covered, ask carers: How has supporting someone with cGvHD impacted on your own life, day-to-day and overall? Follow up with enquiry about psychological/emotional, social, financial impact
- If applicable, sensitively ask carers of patients who have died about the patient's end of life experiences and impact of this on their quality of life

Experience of GvHD symptoms prior to diagnosis

- When you were initially diagnosed with cGvHD, what were your expectations of how this condition might affect your life and how treatment would help? Consider:
    - Expected prognosis/impact on ability to live a 'normal' life
    - Expectation/hope of treatment benefit

Quality of life impact of cGvHD care

- [Interviewer to prioritise covering all previous sections in full and only cover this section if time permits]
- Thinking specifically about the management of cGvHD, which healthcare professionals do you see? How often do you see them? How, if at all, does needing to attend appointments and see healthcare professionals impact on your life?
- Thinking about the impacts you have described cGvHD having on your life (e.g. psychological, social, physical, economic), how, if at all, does the healthcare system support you in managing these?
    - What do you tell the health professionals managing your cGvHD about your symptoms and how cGvHD affects you on a daily basis? Are there things that you don't tell them or find hard to tell them?
    - Are there any issues affecting your quality of life which you would like more support with?
- What advice or support (if any) do health professionals provide you with relating to:
    - Managing symptoms/effect of cGvHD

    - Managing side effects of treatments for cGvHD
    - Managing the psychological/emotional impact of cGvHD
- What were your expectations for the treatment you're currently receiving? How well have those expectations been met? What, if anything, would you like to improve about your current treatment? How, if at all, has this impacted living with cGvHD?
- To what extent, if at all, have you ever wanted to change treatments in the past? If so, what has made you keen to change treatment? What, if anything, would encourage you to consider trying a new treatment?
    - Explore fully the outcomes the patient cares about in terms of their decision making around treatment for cGvHD, and understanding of the drivers for treatment escalation from their perspective

Quantitative survey questions

Carer A = Are currently or have recently been the at-home carer (family member or friend) of someone who currently has GvHD following a donor stem cell transplant

Carer B = Was previously the at-home carer (family member or friend) of someone who had GvHD following a donor stem cell transplant, who has since died

Background

Question 1

- Carer A: What is the age of the person you are caring for?
- Carer B: What age was the person you were caring for when they died?

Question 2

- Patient: At what age did you have the donor stem cell transplant that related to your cGvHD?
- Carer A: At what age did the person you care for have the donor stem cell transplant that related to their cGvHD?
- Carer B: At what age did the person you cared for have the donor stem cell transplant that related to their cGvHD?

Question 3

- Patient: And at what age were you told by a doctor or nurse that you had cGvHD?
- Carer A: And at what age was the person you care for told by a doctor or nurse that they had cGvHD?
- Carer B: And at what age was the person you cared for told by a doctor or nurse that they had cGvHD?

Question 4

- Patient: What condition led to you receiving a donor stem cell transplant?
- Carer A: What condition led to the person you care for receiving a donor stem cell transplant?

- Carer B: What condition led to the person you cared for receiving a donor stem cell transplant?

Respondents asked to choose one option from the list below
Acute myeloid leukemia (AML)
Chronic myeloid leukemia (CML)
Acute lymphocytic leukemia (ALL)
Chronic lymphocytic leukemia (CLL)
Acute lymphoblastic leukemia (ALL)
Acute promyelocytic leukemia (APL)
Multiple myeloma
Aplastic anaemia
Refractory anaemia
Myelofibrosis
Myelodysplasia/other myelodysplastic disorder
Hairy cell leukemia (HCL)
Large granular lymphocytic (LGL) leukemia
Prolymphocytic leukemia (PLL)
Polycythaemia vera (PV)
Essential thrombocythaemia (ET)
Hodgkin lymphoma
Non-hodgkin lymphoma
Amegakaryocytosis/congenital thrombocytopenia
Immune thrombocytopenia
Paroxysmal nocturnal haemoglobinuria (PNH)
Familial hemophagocytic lymphohistiocytosis (fHLH)
Osteoporosis
Wiskott-Aldrich syndrome
Other (specify)
Not sure/don’t know

Pre- and post-diagnosis awareness of cGvHD

Question 5

- Patient: Please think back to before you had your stem cell transplant. Which of the following statements best describes your awareness of the possibility of developing cGvHD at that time?
- Carer A: Please think back to before the person you care for had their stem cell transplant. Which of the following statements best describes their awareness of the possibility of developing cGvHD at that time?
- Carer B: Please think back to before the person you cared for had their stem cell transplant. Which of the following statements best describes their awareness of the possibility of developing cGvHD at that time?

Respondents asked to choose one option from the list below
Fully aware
Mostly aware
Somewhat aware
Not aware at all
For carer A & B only: Not sure

Question 6

- Patient: To what extent do you agree or disagree with each of the following statements about your awareness of cGvHD before you were diagnosed?
- Carers: To what extent do you agree or disagree with each of the following statements about their awareness of cGvHD before they were diagnosed?

Respondents asked to state whether they ‘Agree strongly,’ ‘Agree slightly,’ ‘Neither agree nor disagree,’ ‘Disagree slightly,’ or ‘Disagree strongly;’ Carers were also given the option to select ‘Not applicable/not sure’	
01	Patient: I didn’t know how bad GvHD could be before I was diagnosed<br>Carers: They didn’t know how bad cGvHD could be before they were diagnosed

02	Patient: When I had my stem cell transplant, I wasn't aware that I might suffer with cGvHD long term<br>Carers: When they had their stem cell transplant, they were not aware that they might suffer with cGvHD long term
03	Patient: I knew developing cGvHD was a possibility, but when I was preparing for my stem cell transplant it wasn't something I wanted to think about<br>Carers: They knew developing cGvHD was a possibility, but when they were preparing for their stem cell transplant it wasn't something they wanted to think about

----

Question 7

- Patient: To what extent do you agree or disagree with each of the following statements about your awareness of cGvHD when you were first diagnosed?
- Carer A: To what extent do you agree or disagree with each of the following statements about the person you care for's awareness of cGvHD when they were first diagnosed?
- Carer B: To what extent do you agree or disagree with each of the following statements about the person you cared for's awareness of cGvHD when they were first diagnosed

Respondents asked to state whether they 'Agree strongly,' 'Agree slightly,' 'Neither agree nor disagree,' 'Disagree slightly,' or 'Disagree strongly;' Carers were also given the option to select 'Not applicable/not sure'	
01	Patient: When I was first told I had cGvHD, I found it difficult to come to terms with<br>Carers: When they were first told they had cGvHD, they found it difficult to come to terms with
02	Patient: I was fully aware of all treatment options available when I was first diagnosed with cGvHD<br>Carers: They was fully aware of all treatment options available when they were first diagnosed with cGvHD
03	Patient: When I was first told I had cGvHD, I expected treatment would be effective

	Carers: When they were first told they had cGvHD, they expected treatment would be effective
04	Patient: When I was first told I had cGvHD, I didn't appreciate how long I could be experiencing symptoms for<br>Carers: When they were first told they had cGvHD, they didn't appreciate how long they could be experiencing symptoms for
05	Patient: When I was first told I had cGvHD it was easy for me to access support to help me manage my symptoms<br>Carers: When they were first told they had cGvHD it was easy for them to access support to help them manage symptoms
06	All: It was a shock to receive a diagnosis of cGvHD

----

cGvHD symptoms

Question 8

- Patient: Which of the following physical symptoms have you ever experienced as a result of cGvHD?
- Carer A: Which of the following physical symptoms has the person you care for ever experienced as a result of cGvHD?
- Carer B: Which of the following physical symptoms did the person you cared for ever experience as a result cGvHD?

Respondents asked to select all that apply	
Skin - A	Skin rash/blotches
	Red, inflamed, sun-burn like skin
	Raised bumps on skin
	Flat, thickened areas of skin (plaques) that are flaky or scaly
	Itchy skin
	Painful/sore/dry skin
	Skin that's sensitive to the sun
	Skin thickening, hardening or tightening (scleroderma)

	Nail changes (e.g. texture, hard / brittle nails)
	Difficulty sweating
	Sensitivity to heat or cold
	Other skin symptoms
Eyes - B	Dry/itchy eyes
	Gritty eyes/feeling there’s something in your eyes
	Painful eyes
	Eyes sensitive to light
	Red/swollen eyelids/blepharitis
	‘Spider veins’ on eyelid
	Other eye symptoms
Mouth - C	Dry mouth
	Red/sore mouth
	Mouth ulcers
	Sensitive mouth (e.g. can’t eat spicy/hot food)
	Any other mouth symptoms
Lungs - D	Shortness of breath/breathlessness
	Pneumonia
	Inflammation in the airways of your lungs (e.g. bronchiolitis obliterans)
	Continuous cough
	Any other lung symptoms
Genitals - E	Vaginal dryness/burning/itching
	Narrowing of the vagina
	Painful sexual intercourse or bleeding after sex
	Pain passing urine
	Decreased libido/sex drive
	Redness/rash/ulcers on genitals
	Itching of skin on genitals
	Inability to ejaculate
	Splitting/tearing/loss/scarring of skin of genitals
	Other genital symptoms

Joints - F	Stiff joints
	Decreased range of movement in joints/problems straightening arms or legs
	Skin tightening/hardening around joints
	Other joint symptoms
Musculoskeletal - G	Muscle loss/wastage
	Muscle cramps
	Muscle weakness
Hair - H	Hair loss
	Changes in hair colour or consistency
Gut / stomach / bowel - I	Difficulty swallowing
	Nausea and/or vomiting
	Diarrhoea
	Narrowing of the food pipe/oesophagus
	Acid reflux
	Ulcers in colon/intestine
	Lack of appetite/feeling full after not eating very much
	Stomach pain or swelling
	Blood in stool
	Difficulty digesting food/indigestion
	Other gut/stomach/bowel symptoms
Liver - J	Liver cirrhosis/scarring (late-stage liver disease)
	Yellow skin/eyes/jaundice
Kidney - K	Kidney damage/disease/failure
Infections - L	Prone to frequent infections (e.g. colds, flu, fungal infections, stomach bugs)
	Chest infections
	Urinary tract infections
	Skin infections
	Sepsis
General - M	Weight loss
	General pain

	Fatigue/tiredness
	Fluid retention (edema)/swelling of body parts such as ankles
Other (specify)	
None of the above	

Question 9

- Patient: For each symptom you have experienced due to cGvHD, please tell us:
    - How often is/was the symptom experienced?
    - How much impact it has/had on your daily life?
- Carer A: For each symptom the person you care for has experienced due to cGvHD, please tell us:
    - How often is/was the symptom experienced?
    - How much impact it has/had on their daily life?
- Carer B: For each symptom the person you cared for has experienced due to cGvHD, please tell us:
    - How often is/was the symptom experienced?
    - How much impact it has/had on their daily life?

Respondents asked to choose an option for each symptom group named from the previous question						
	Every day/ most days/ always have this	Frequently (experience this often, but not every day)	It flares up intermittently (can go for weeks or months without experiencing this)	It flares up rarely (can go several months or years without experiencing this)	It only flares up when triggered by something (e.g. an infection, stop in medication such as steroids or	Carers only: Not sure

					immunosuppressants)	
**SHOW GROUP (A-L) NAME SELECTED FROM PREVIOUS Q8 symptoms**	01	02	03	04	05	06

Respondents asked to choose an option for each symptom group named from the previous question					
	High impact	Moderate impact	Low impact	No impact at all	Carers only: Not sure
**SHOW GROUP (A-L) NAME SELECTED FROM PREVIOUS Q8 symptoms**	01	02	03	04	05

Question 10

- Patient: Which of the following cGvHD symptoms are you experiencing at the moment?
- Carer A: Which of the following cGvHD symptoms is the person you're caring for experiencing at the moment?
- (Carer B not asked)

Select from list of previously selected symptoms at Q8 or:

Not currently experiencing any of these symptoms
Not sure/don't know

Question 11

- To what extent do you agree or disagree with each of the following statements about cGvHD symptoms?

Respondents asked to state whether they 'Agree strongly,' 'Agree slightly,' 'Neither agree nor disagree,' 'Disagree slightly,' or 'Disagree strongly;' carers were also given the option to select 'Not applicable/not sure'	
01	Patient: I waited longer than I should have to talk to a doctor about my symptoms, because I thought some GvHD is a good sign<br>Carer A: The person I care for waited longer than they should have to talk to a doctor about symptoms, because they thought some GvHD is a good sign<br><br>(Carer B not asked)
02	There should be more information for patients about what to look out for when you start developing cGvHD
03	It was clear that new symptoms were cGvHD, before speaking to a doctor
04	Patient: My partner or family members often help me identify symptoms of cGvHD<br>Carer A: I or other members of the family help them identify symptoms of cGvHD<br>Carer B: I, or other members of the family helped them identify symptoms of cGvHD
05	Patient: I need a lot of support from my partner or family to help manage my cGvHD<br>Carer A: The person I care for needs a lot of support from me or family to help manage their cGvHD<br>Carer B: The person I cared for needed a lot of support from me or family to help manage their cGvHD

Treatment and healthcare

Question 12

- Patient: Which of the following additional treatments or medicines, if any, have you ever taken to help control or prevent cGvHD symptoms, to treat conditions resulting from cGvHD, or control side effects caused by other treatments?
- Carer A: Which of the following additional treatments or medicines, if any, have they ever taken to help control or prevent cGvHD symptoms, to treat conditions resulting from cGvHD, or control side effects caused by other treatments?
- Carer B: Which of the following treatments, additional treatments or medicines, if any, did they take to help control or prevent cGvHD symptoms, to treat conditions resulting from cGvHD, or control side effects caused by other treatments?

Respondents were asked to tick all that apply
Medicines for digestion e.g. diarrhoea, heartburn
Medicines for increasing appetite
Hormonal treatments e.g. hormone replacement therapy, testosterone, hormonal patches or gels
Medicines for nausea/vomiting
Anti-depressants or anti-anxiety medication
Anti-viral medicines
Antibiotics
Medicines for bladder control
Medicines for cholesterol
Medicines for breathing/asthma symptoms e.g. inhalers
Antihistamines
Medicines for osteoporosis/brittle bones
Medicines to help insomnia/sleeping
Other (specify)
Not taking any additional treatments or medicines
Not sure/don’t know

Question 13

- Patient: And thinking about all the medicines that you have ever taken for cGvHD, which of these medicines are you currently taking now?
- Carer A: And thinking about all the medicines that they have ever taken for cGvHD, which of these medicines are they currently taking now?
- (Carer B not asked)

Question 14

- Patient: What side effects, if any, have you experienced as a result of the treatments received?
- Carer A: What side effects, if any, have they experienced as a result of the treatments received?
- Carer B: What side effects, if any, did they experience as a result of the treatments received?

Respondents were asked to tick all that apply	
Skin - A	Skin rash/blotches
	Itchy skin
	Dry skin
	Painful/sore/dry skin
	Nail changes (e.g. texture, hard/brittle nails)
	Thin/easily damaged skin
	Acne/rosacea (redness)
	Skin that is sensitive to the sun
	Other skin side effects
Eyes - B	Cataracts
	Dry/itchy eyes
	Other eye side effects
Mouth - C	Dry mouth
	Changes in taste
	Red/sore mouth
	Mouth ulcers

	Sensitive mouth (for example, can't eat spicy/hot food)
	Other mouth side effects
Lungs - D	Shortness of breath/breathlessness
	Pneumonia
	Asthma
	Chest pain
	Continuous cough
	Other lung side effects
Genitals - E	Urgent need to pass urine
	Low libido/sex drive
	Other side effects affecting the genitals
Joints and bones - F	Osteoporosis/brittle bones/fractures
	Joint pain
	Other joint/bone side effects
Muscles - G	Muscle loss/wastage
	Muscle pain/cramps/tremors
	Muscle weakness
	Lupus
	Other muscle side effects
Hair - H	Hair loss
	Increased hair growth
	Other hair changes
Gut / stomach / bowel - I	Difficulty swallowing
	Nausea and/or vomiting
	Diarrhoea
	Constipation
	Acid reflux
	Lack of appetite/feeling full after not eating very much
	Stomach pain or swelling
	Weight gain
	Weight loss
	Other stomach or bowel side effects

Liver - J	Liver cirrhosis/scarring (late-stage liver disease)
	Yellow skin/eyes/jaundice
Kidney - K	Kidney damage/disease
Infections - L	Prone to/frequent infections (e.g. colds, flu, fungal infections, stomach bugs)
	Chest infections
	Urinary tract infections
	Thrush
	Skin infections
	Gut infections/stomach bugs
	Sepsis
	Shingles
	Infections to portacath/subcutaneous port/Hickman line (where a thin tube is inserted into your veins under the skin to give medicine more easily. It can stay in place for many months)
	Other infections
Heart / circulatory system - M	Heart problems
	High cholesterol
	High blood pressure
	Anaemia (low levels of red blood cells)
	Other heart or blood side effects
Sensory system - N	Headaches
	Insomnia/difficulty sleeping
	Dizziness
	Tinnitus/ringing in ears
	Other sensory side effects
O	Fevers/chills/flu-like symptoms
	General pain
	Fatigue/tiredness
	Fluid retention (edema)/swelling of body parts such as ankles
	Round, puffy face/moon-face

	A build-up of fat on the back of your neck and shoulders (known as a ‘buffalo hump’)
Diabetes	
Other (specify)	
No side effects experienced	
Not sure/don’t know	

Question 15

- Patient: For each of the side effects you experienced as a result of treatment, how did this impact on your day-to-day life?
- Carer A: For each of the side effects they experienced as a result of treatment, how has this impacted their day-to-day life?
- Carer B: For each of the side effects they experienced as a result of treatment, how did this impact their day-to-day life?

Respondents asked to choose an option for each side effect named from the previous question					
	High impact	Moderate impact	Low impact	No impact at all	Carers only: Not sure
**SHOW GROUP (A-L) NAME SELECTED FROM PREVIOUS Q14 side effects**	01	02	03	04	05

Question 16

- Patient: Which of the following surgical procedures or operations have you had as a result of your cGvHD?

- Carer A: Which of the following surgical procedures or operations have they had a s a result of their cGvHD?
- Carer B: Which of the following surgical procedures or operations did they have as a result of their cGvHD?

Respondents asked to select all that apply
Feeding tube
Grafts on eyes
Cataract surgery
Surgery to correct blocked bowel or hole in the large intestine (bowel perforation or obstruction) or bleed (haemorrhage)
Insertion of a portacath/subcutaneous port/Hickman line (where a thin tube is inserted into your veins under the skin to give medicine more easily. It can stay in place for many months)
Other surgery or procedure (specify)
No procedures or surgeries

Question 17

- To what extent do you agree or disagree with each of the following statements about treatment for cGvHD?

Respondents asked to state whether they 'Agree strongly,' 'Agree slightly,' 'Neither agree nor disagree,' 'Disagree slightly,' or 'Disagree strongly;' carers were also given the option to select 'Not applicable/not sure'	
01	Patient: I struggle to remember what each of my treatments is for<br>Carer A: The person I care for struggles to remember what each of their treatments is for<br>Carer B: The person I cared for struggled to remember what each of their treatments was for
02	Treatments for cGvHD do not work very well

03	Patient: I have been prescribed treatments which cause interactions with a treatment I am already taking<br>Carer A: The person I care for has been prescribed treatments which cause interactions with a treatment they are already taking<br>Carer B: The person I cared for was prescribed treatments which caused interactions with a treatment they were already taking
04	The side effects caused by treatments can be worse than the cGvHD itself
05	Patient: The treatment regimen for my cGvHD is so complicated that sometimes I struggle to leave the house<br>Carer A: The treatment regimen for cGvHD is so complicated that sometimes the person I care for can struggle to leave the house<br>Carer B: The treatment regimen for cGvHD is so complicated that sometimes the person I cared for would struggle to leave the house
06	There is not enough information about different treatment options for cGvHD
07	There is not enough information about ways to self-manage cGvHD
08	Patient: I worry about having to go to hospital to receive my cGvHD treatment<br>Carer A: The person I care for worries about having to go to hospital to receive cGvHD treatment<br>Carer B: The person I cared for worried about having to go to hospital to receive cGvHD treatment

----

ECP

Question 18

- Patient: Which of the following best describes your experiences of ECP treatment?
- Carer A: Which of the following best describes the person you care for's experiences of ECP treatment?
- Carer B: Which of the following best describes the person you cared for's experiences of ECP treatment?

Respondents asked to choose one option from the list below
Currently receiving ECP (not shown to carer B)
Waiting to start treatment (not shown to carer B:
Received ECP treatment in the past
Offered ECP treatment but did not go ahead (for whatever reason)
Not been offered ECP
Not sure

Question 19

- Patients currently receiving ECP: How often do you attend appointments for ECP?
- Patients who previously received ECP: When you stopped, how often did you attend appointments for ECP?
- Carer A of patients currently receiving ECP: How often do they attend appointments for ECP?
- Carer A of patients who previously received ECP: When they stopped, how often did they attend appointments for ECP?
- Carer B of patients who received ECP: How often were they attending appointments for ECP?

Respondents asked to choose one option from the list below
Multiple times a week
Once a week
Multiple times a month
Once a month
A few times in a year
Once a year
Less than once a year
Not sure

Question 20

- Patients currently receiving ECP: How long have you been receiving ECP?
- Patients who previously received ECP: How long did you receive ECP for?
- Carer A of patients currently receiving ECP: How long have they been receiving ECP?
- Carer A of patients who previously received ECP: How long did they receive ECP for?
- Carer B of patients who received ECP: How long did they receive ECP for?

Question 21

- To what extent do you agree or disagree with each of the following statements?

Respondents asked to state whether they 'Agree strongly,' 'Agree slightly,' 'Neither agree nor disagree,' 'Disagree slightly,' or 'Disagree strongly;' carers were also given the option to select 'Not applicable/not sure'	
01	Patient/Carer A: Attending regular ECP appointments has not caused any financial issues<br>Carer B: Attending regular ECP appointments did not cause any financial issues
02	Patient: I found it easy to take time out and fit ECP into my schedule<br>Carers: They found it easy to take time out and fit ECP into their schedule
03	ECP treatment causes severe fatigue
04	Sun-sensitivity from ECP makes it difficult to go outside
05	It is easy to co-ordinate ECP appointments

Question 22

- Patient who previously received ECP: Why have you stopped treatment with ECP?
- Patient who was offered ECP but did not go ahead: Why did you decide not to have treatment with ECP?
- Carer A of patients who previously received ECP: Why have they stopped treatment with ECP?
- Carers A + B of patients who were offered ECP but did not go ahead: Why did they decide not to have treatment with ECP?
- (Carer B of patients who previously received ECP not asked)

Respondents asked to select all that apply
The doctor said it would be better to try a different treatment
Travel to the hospital was too difficult
Too much time commitment
Travel to the hospital cost too much money
Concerns about side effects

Difficulty accessing veins / difficulties with port or line access
Fatigue / tiredness
Side effects
Treatment was successful
Other reason (specify)
Not sure / don't know

HCPs

Question 23

- Patient: Which of the following healthcare professionals have you ever seen in relation to your cGvHD?
- Carer A: Which of the following healthcare professionals have they ever seen in relation to their cGvHD?
- Carer B: Which of the following healthcare professionals did they ever see in relation to their cGvHD?

Respondents asked to select all that apply
Haematologist (blood)
Dermatologist (skin)
Gynaecologist (genital)
Urologist (urinary)
Gastroenterologist (gut/bowel)
Hepatoligst (liver)
Ophthalmologist (eyes)
Pulmonologist (lungs)
Rheumatologist (musculoskeletal)
Psychologist / psychiatrist
Cardiologist (heart)
Neurologist (brain / spine / nerves)
Pain specialist

GP
Other (specify)
None of the above
Not sure / don’t know

Question 24

- Patient: On average, how frequently do you attend medical appointments or see healthcare professionals about your cGvHD or issues relating to your cGvHD?
- Carer A: On average, how frequently does the person you care for attend medical appointments or see healthcare professionals about their cGvHD or issues relating to their cGvHD?
- Carer B: On average, how frequently did the person you care for attend medical appointments or see healthcare professionals about their cGvHD or issues relating to their cGvHD?

Respondents asked to choose one option from below
Multiple times a week
Once a week
Multiple times a month
Once a month
A few times in a year
Once a year
Less than once a year
Not sure

Question 25

- Patient: On average, how often do you have unplanned or emergency hospital visits or hospital stays (including going to A&E and/or being admitted to a ward), as a result of your cGvHD or complications related to it?

- Carer A: On average, how often does the person you care for have any unplanned or emergency hospital visits or hospital stays (including going to A&E and/or being admitted to a ward), as a result of their GvHD or complications related to it?
- Carer B: On average, how often did the person you care for have unplanned or emergency hospital visits or hospital stays (including A&E and/or being admitted to a ward), as a result of their cGvHD or complications related to it?

Respondents asked to choose one option from below (and to not include routine or planned hospital appointments)
Multiple times a week
Once a week
Multiple times a month
Once a month
A few times in a year
Once a year
Less than once a year
Not sure

Question 26

- To what extent do you agree or disagree with each of the following statements?

Respondents asked to state whether they 'Agree strongly,' 'Agree slightly,' 'Neither agree nor disagree,' 'Disagree slightly,' or 'Disagree strongly;' carers were also given the option to select 'Not applicable/not sure'	
01	Healthcare professionals are generally well informed about cGvHD
02	There needs to be more communication between the different healthcare professionals that manage cGvHD
03	Doctors fully understand how severe cGvHD symptoms can be
04	Patient: I found it easy to take time out and fit all my doctors appointments into my schedule

	Carers: They found it easy for them to take time out and fit all their doctors appointments into their schedule
05	Patient/Carer A: Travelling to appointments for cGvHD is straightforward<br>Carer B: Travelling to appointments for cGvHD was straightforward
06	Patient/Carer A: Attending regular doctors appointments has not created any financial problems<br>Carer B: Attending regular doctors appointments did not create any financial problems
07	Appointments with different doctors are always well organised
08	There should be just one doctor to manage all of cGvHD

Patient QoL

Question 27

- Patient: Please tell us how much impact cGvHD has on each of these aspects of your daily life
- Carer A: Please tell us how much impact cGvHD has on each of these aspects of the daily life of the person you care for
- Carer B: Please tell us how much impact cGvHD had on each of these aspects of the daily life of the person you cared for

Respondents asked to define the level of impact cGvHD had on the below aspects of their daily life; options were: 'High impact,' 'Moderate impact,' 'Low impact,' or 'No impact at all;' Carers were also given the option to select 'Not sure'	
01	Everyday mobility and physical activity
02	Eating and drinking
03	Exercise
04	Carrying out simple daily tasks e.g. preparing food, getting dressed
05	Being independent
06	Enjoying life
07	Spending quality time with family/friends
08	Self-confidence
09	Sleep

10	Personal hygiene e.g. washing, brushing teeth
11	Aspirations for the future
12	Ability to work/study
13	Managing the finances
14	Mental health
15	Planning for the future
16	Intimate relationships
17	Hobbies
18	Appearance
19	Management of the condition that lead to the stem cell transplant

----

Question 28a

- Patients: Please now think about how having cGvHD has impacted on your working life. Which of the following, if any, apply to you?
- Carer A: Please now think about how having cGvHD has impacted the working life of person you care for. Which of the following apply to them?
- Carer B: Please now think about how having cGvHD impacted on the working life of the person you cared for. Which of the following applied to them?

Respondents asked to tick all that apply
Changed jobs/career
Stopped work completely/retired early
Changed working patterns (e.g. number of hours/shift patterns/role)
Delayed return to work
Taken time off work/sick leave/extended absence
None of the above/no impact on working life
Not applicable/was not working prior to having cGvHD
Not sure/don’t know

Question 28b

- Patients: And which of the following, if any, apply to the impact cGvHD on the working life of your partner, family, or those who care for you?
- Carer A: And how, if at all, has caring for someone with cGvHD impacted on your working life?
- Carer B: And how, if at all, did caring for someone with cGvHD impact on your working life?

Respondents asked to tick all that apply
Changed jobs/career
Stopped work completely/retired early
Changed working patterns (e.g. number of hours/shift patterns/role)
Delayed return to work
Taken time off work/sick leave/extended absence
None of the above/no impact on working life
Not applicable/was not working prior to having cGvHD
Not sure/don't know

Question 29

- To what extent do you agree or disagree with the following statements?

Respondents asked to state whether they 'Agree strongly,' 'Agree slightly,' 'Neither agree nor disagree,' 'Disagree slightly,' or 'Disagree strongly;' carers were also given the option to select 'Not applicable/not sure'	
01	Patient: I have had difficulties accessing the welfare benefits I am entitled to<br>Carer A: They have had difficulties accessing the welfare benefits they are entitled to<br>Carer B: They had difficulties accessing the welfare benefits they were entitled to
02	Patient: I (or my partner/family) have experienced discrimination from my employer due to my cGvHD<br>Carer A: They (or their partner/family) have experienced discrimination from their employer due to cGvHD<br>Carer B: They (or their partner/family) experienced discrimination from their employer due to cGvHD

EQ-5D and VAS (only patients and carer A completed the EQ-5D; all respondents completed VAS)

- Performed in accordance with instructions found in the EQ-5D-5L representation and VAS guidelines

----

Patient mental health

Question 30

- Patient: Please think about your treatment journey overall, from the time you were diagnosed with [condition stated in Q4] through to having your stem cell transplant, through to diagnosis with cGvHD.
    - On a scale of 0 to 10, where 0 is very negative and 10 is very positive, how did you feel at each of the following points.
- Carer A: Please think about the treatment journey overall, from the time the person you care for was diagnosed with [condition stated in q4] through to having their stem cell transplant, through to diagnosis with cGvHD.
    - On a scale of 0 to 10, where 0 is very negative and 10 is very positive, how did you feel at each of the following points.
- Carer B: Please think about the treatment journey overall, from the time the person you cared for was diagnosed with [condition stated in Q4] through to having their stem cell transplant, through to diagnosis with cGvHD.
    - On a scale of 0 to 10, where 0 is very negative and 10 is very positive, how did you feel at each of the following points.

Respondents asked to rate the stages in their cGvHD on a scale of 0–10, 0 being very negative and 10 being very positive	
	**Patient**
01	Being diagnosed with [condition stated in Q4]
02	Initial treatment for [condition stated in Q4]
04	Being told you need a stem cell transplant
05	Being told you had found a suitable donor
06	Immediately after having your stem cell transplant
07	When you first developed symptoms of GvHD
08	When you were told you had cGvHD long term
09	Today

Respondents asked to rate the stages in their patients' cGvHD on a scale of 0–10, 0 being very negative and 10 being very positive	
	**Carers**
01	Being diagnosed with [condition stated in Q4]
02	Initial treatment for [condition stated in Q4]
04	Being told they need a stem cell transplant
05	Being told they had found a suitable donor
06	Immediately after having their stem cell transplant
07	When they first developed symptoms of GvHD
08	When they were told they had cGvHD long term
09	Today [Not shown to carer B]

Question 31

- Patient: Which of the following emotions, if any, have you experienced in connection with your cGvHD?
- Carers: Which of the following emotions, if any, have you experienced the most when supporting and caring for someone with cGvHD?

Respondents ask to select up to five emotions, selecting the most common emotion first
Stressed
Anxious/worried
Low mood/depressed
Guilty
Angry/annoyed
Frustrated
Hopeless
Isolated
Apathy
Embarrassed
Traumatized
Trapped/restricted

Unfulfilled
Out of control
Disappointed
None of these

Question 32

- To what extent do you agree or disagree with each of the following statements?

Respondents asked to state whether they 'Agree strongly,' 'Agree slightly,' 'Neither agree nor disagree,' 'Disagree slightly,' 'Disagree strongly,' 'Not applicable,' or 'Skip this question'	
01	I changed how I live my life due to infection risks (e.g. avoiding enclosed public spaces like supermarkets or cinemas, shielding, avoiding public transport, avoiding meeting people inside)
02	Patient: I have been able to reach out for support when I needed it<br>Carer A: The person I care for has been able to reach out for support when needed<br>Carer B: The person I cared for was able to reach out for support when needed
03	People with cGvHD need more emotional support than is currently on offer
04	Patient: Having cGvHD has impacted my mental health<br>Carer A: Having cGvHD has impacted their mental health<br>Carer B: Having cGvHD impacted their mental health
05	Worrying about cGvHD keeps me awake at night<br>[Not shown to carer B]
06	Patient: I have missed out on major life events with family or friends because of my cGvHD<br>Carer A: The person I care for has missed out on major life events with family or friends because of their cGvHD<br>Carer B: The person I cared missed out on major life events with family or friends because of their cGvHD
07	Patient: My self-confidence is not affected by having cGvHD<br>Carer A: Having cGvHD has had no effect on their self-confidence<br>Carer B: Having cGvHD had no effect on their self-confidence

08	Patient: I sometimes feel I am a burden to others because of my cGvHD<br>Carer A: The person I care for sometimes feels they are a burden to others because of their cGvHD<br>Carer B: The person I cared for sometimes felt they were a burden to others because of their cGvHD
09	Patient: I feel worse with cGvHD than when I had my previous condition<br>Carer A: They feel worse with cGvHD than when they had their previous condition<br>Carer B: They felt worse with cGvHD than when they had their previous condition
10	Patient: I am able to live my life how I want to, despite my cGvHD<br>Carer A: The person I care for is able to live their life how they want to, despite their cGvHD<br>Carer B: The person I cared for was able to live their life how they wanted to, despite their cGvHD
11	Patient: My friends and family really understand how cGvHD makes me feel<br>Carer A: The person I care for thinks friends and family really understand how cGvHD makes them feel<br>Carer B: The person I cared for thought friends and family really understood how cGvHD made them feel
12	Patient: I have come to terms with the fact my appearance has changed as a result of my cGvHD<br>Carer A: They have come to terms with the fact their appearance has changed as a result of cGvHD<br>Carer B: They came to terms with the fact their appearance changed as a result of cGvHD
13	Patient: I have come to terms with the fact cGvHD is unpredictable and you cannot be sure when a new symptom<br>will arise<br>Carer A: The person I care for has come to terms with the fact cGvHD is unpredictable and you cannot be sure when a new symptom will arise<br>Carer B: The person I cared for came to terms with the fact cGvHD is unpredictable and you could not be sure when a new symptom would arise
14	Patient: I am more worried about my cGvHD than I am about a possible relapse of my [insert condition from Q3]

	Carer A: The person I care for is more worried about their cGvHD than they are about a possible relapse of their [insert condition from Q3]<br>Carer B: The person I cared for was more worried about their cGvHD than they were about a possible relapse of their [insert condition from Q3]
15	Patient: After my stem cell transplant I expected my life to return to normal<br>Carers: After their stem cell transplant they expected their life to return to normal

----

Question 33

- Patient: Which of the following statements describe the mental health support you have been offered in relation to your cGvHD?
- Carer A: Which of the following statements describe the mental health support that has been offered to the person you care for in relation to their cGvHD?
- Carer B: Which of the following statements describe the mental health support that was offered to the person you cared for in relation to their cGvHD?

Respondents asked to select all that apply
Offered mental health support through the NHS and accepted it
Offered mental health support through the NHS but did not accept it
Not offered any mental health support through the NHS
Accessed/funded mental health support privately (not through the NHS)
Not sure

Carer QoL

Question 34

- Carer A: Please tell us how much impact cGvHD has on each of these aspects of your daily life, as a carer of someone with cGvHD
- Carer B: Please tell us how much impact cGvHD had on each of these aspects of your daily life, as a carer for someone with cGvHD

Respondents asked to define the level of impact cGvHD had on the below aspects of their daily life; options were: 'High impact,' 'Moderate impact,' 'Low impact,' or 'No impact at all;' carers were also given the option to select 'Not sure'	
01	Eating and drinking
02	Exercise
03	Being independent
04	Enjoying life
05	Spending quality time with family / friends
06	Self-confidence
07	Sleep
08	Aspirations for the future
09	Ability to work / study
10	Managing the finances
11	Mental health
12	Planning for the future
13	Intimate relationships
14	Hobbies
15	Management of the condition that lead to the stem cell transplant

----

Question 35

- Carers: To what extent do you agree or disagree with each of the following statements?

Respondents asked to state whether they 'Agree strongly,' 'Agree slightly,' 'Neither agree nor disagree,' 'Disagree slightly,' 'Disagree strongly,' or 'Skip this question'	
01	I have been reluctant to reach out for support when needed
02	Carers for people with cGvHD need more emotional support than is currently on offer
03	Carer A: I feel like when caring for someone with cGvHD my own life is on hold<br>Carer B: I felt like when I was caring for someone with cGvHD that my own life was on hold
04	Carer A: I try to hide how much caring for someone with cGvHD impacts me<br>Carer B: I tried to hide how much caring for someone with cGvHD impacted me

05	Supporting someone with cGvHD has been much harder than expected
06	Caring for someone with cGvHD has negatively impacted my mental health
07	Carer A: I feel there is enough information available to support me in doing a good job as a carer<br>Carer B: I feel there was enough information available to support me in doing a good job as a carer
